# Crown Work and English Tube Teeth

**Published:** 1886-01

**Authors:** Lawrence Vanderpant

**Affiliations:** Orange, N. J.


### CROWN WORK AND ENGLISH TUBE TEETH.

BY DR. LAWRENCE VANDERPANT, ORANGE, N. J.

The interest created by the publication of my two articles on this subject is certainly wide-spread, if not universal, for I have had correspondence from remote places, the existence of which an ordinary map does not reveal. My reply has been a postal card with the intimation, "*Vide* next number of the INDEPENDENT PRACTITIONER," so I will now crave a short space.

1st. A puzzling error occurs in the November article, page 583, first line: for "pickling" read "pitching," and the context will explain the meaning of the term; it is an old and ordinary laboratory one.

2d. As a general reply to enquirers, I will briefly epitomize the matter as regards crown or pivot work.

(*a.*) Tube teeth are not capable of universal application, even as regards the incisors and cuspids; in rare instances they will be too thick to articulate.

(*b.*) The object of trimming the portion of the pin to be affixed to the root, is that a union may be formed with the amalgam used for securement.

(*c.*) It adds materially to the value of this work to slightly screw the pin into the apex of the cavity, as by so doing the pin can be accurately bent to requirement; it will maintain its position during the plugging of the amalgam, and it will not draw in the final adjustment of the tube tooth-crown.

(*d.*) The Herbst method is advantageous in filling the root. An appropriate instrument will suggest itself, as for example, a thick broken needle.

(*e.*) A solution of gutta-percha, with the floss silk, will make a good cement to fasten the tube tooth to the pin.

(*f.*) The composition (the body) of the teeth of Messrs. Ash & Sons is so much denser, harder, and stronger than that of other descriptions, that they can be reduced, for necessary articulation, to a very considerable degree, by grinding, and will still possess the solidity requisite for practical purposes.

The possession, on the part of the operator, of a small mental capacity to conceive, and a great deal of skill to execute, will add materially to success in this, as well as in many other dental operations.

